# CLEC2 and CLEC5A: Pathogenic Host Factors in Acute Viral Infections

**DOI:** 10.3389/fimmu.2019.02867

**Published:** 2019-12-06

**Authors:** Pei-Shan Sung, Shie-Liang Hsieh

**Affiliations:** ^1^Genomics Research Center, Academia Sinica, Taipei, Taiwan; ^2^Institute of Clinical Medicine, National Yang-Ming University, Taipei, Taiwan; ^3^Department of Medical Research, Taipei Veterans General Hospital, Taipei, Taiwan; ^4^Institute for Cancer Biology and Drug Discovery, Taipei Medical University, Taipei, Taiwan

**Keywords:** CLEC2, CLEC5A, Syk, TLR, extracellular vesicle, microvesicle, exosome

## Abstract

The protective roles of endosomal toll-like receptors (TLRs) and cytosolic nucleic acid sensors are well elucidated, but the pathogenic host factors during viral infections remain unclear. Spleen tyrosine kinase (Syk)-coupled C-type lectins (CLECs) CLEC2 and CLEC5A are highly expressed on platelets and myeloid cells, respectively. CLEC2 has been shown to recognize snake venom aggretin and the endogenous ligand podoplanin and acts as a critical regulator in the development and immunothrombosis. Although CLEC2 has been reported to interact with type I immunodeficiency virus (HIV-1), its role in viral infections is still unclear. CLEC5A binds to fucose and mannose moieties of dengue virus membrane glycans, as well as to N-acetylglucosamine (GlcNAc)/N-acetylmuramic acid (MurNAc) disaccharides that form the backbone of *L. monocytogenes* peptidoglycans. Recently, we demonstrated that both CLEC2 and CLEC5A are critical in microbe-induced “neutrophil extracellular trap” (NET) formation and proinflammatory cytokine production. Moreover, activation of CLEC2 by dengue virus (DV) and H5N1 influenza virus (IAV) induces the release of extracellular vesicles (EVs), which further enhance NETosis and proinflammatory cytokine production via CLEC5A and Toll-like receptor 2 (TLR2). These findings not only illustrate the immunomodulatory effects of EVs during platelet-leukocyte interactions, but also demonstrate the critical roles of CLEC2 and CLEC5A in acute viral infections.

## Introduction

The global outburst of severe acute respiratory syndrome coronavirus (SARS-CoV) infection in 2003 inspired our exploration of how a viral infection can activate a massive over-production of cytokines by the host immune system–a phenomenon known as a “cytokine storm”; this results in a severe inflammatory reaction and increases systemic vascular permeability within 2–5 days following viral exposure. The common features of SARS-CoV and other acute viral infections are the early onset of inflammatory reactions with elevated local or systemic vascular permeability, before the adaptive immune system is fully activated. These observations suggest that innate immune responses contribute significantly to the pathogenesis of acute viral infections.

The identification of endosomal TLRs (TLR3, TLR7, TLR8, TLR9) and cytosolic sensors led to the discovery that their activation by viral nucleic acids induces the production of interferons and proinflammatory cytokines. These intracellular nucleic acid receptors/sensors have been defined as “protective host factors” as they are critical for host defense against viral infections. However, the identity and contribution of “pathogenic host factors” to virus-induced severe inflammatory reactions and lethality, and how different viruses cause distinct clinical symptoms, has remained unclear. In contrast to the relatively conserved nature of nucleic acids (DNA and RNA), the proteins and glycans of viral envelopes are various have distinct structures; it is enveloped viruses, such as flavivirus, alphavirus, togavirus, coronavirus, orthomyxovirus, paramyxovirus, rhabdovirus, bunyavirus, and filovirus that are primarily responsible for severe inflammatory reactions following infection. Therefore, we were interested to determine whether viruses activate membrane receptors on myeloid cells induce cytokine storms, and how these receptors contribute to complex cell-cell interactions during acute viral infections. In this review article, we illustrate the critical roles of C-type lectin receptors in innate immunity and discuss the potential of targeting these cell surface receptors in the treatment of acute viral infections.

## Acute Viral Infections

The common feature of acute viral infections is the rapid onset of inflammatory reactions after exposure to the virus. Clinical symptoms range from inapparent and mild fever to severe inflammation and increased endothelial permeability in major organs. Severe acute infections in human typically occur in response to enveloped viruses, such as dengue virus (flaviviridae), chikungunya virus (togaviridae), SARS-CoV (coronaviridae), influenza virus (orthomyxoviridae), measles virus (paramyxoviridae), rabies virus (rhabdoviridae), hanta virus (bunyaviridae), and Ebola virus and Marburg virus (filoviridae). This implies that membrane components may contribute to the severe inflammatory responses.

Acute viral infections begin with host exposure to virus and clinical symptoms become apparent gradually as the virus starts to replicate and innate immune responses are activated. Abundant proinflammatory cytokines production leads to the characteristic symptoms in acute viral infections, including fever, aches, and pain. When host innate immunity fails to contain virus replication and remove infected cells, high fever and massive cytokine production occur in the host, leading to severe local and systemic changes in vascular permeability, functional failure of affected organs and shock syndromes.

It is well-known most of pro-inflammatory cytokines are released from macrophages and severe acute infections are usually associated with the activation of macrophages by enveloped viruses such as dengue virus (1, 2), H5N1 viruses (2–4), and Ebola viruses (5). In addition, activation of neutrophils leads to the formation of neutrophil extracellular traps (NETs), which may further aggravate virus-induced inflammatory reactions. Furthermore, acute viral infections frequently cause thrombocytopenia, and platelet-leukocyte interactions not only regulate inflammatory reactions, but also contribute to the pathogenesis of vascular injury, thrombosis, autoimmune reactions (6–8). However, the molecular mechanisms underlying platelet-mediated immunomodulation are still unclear.

## Nucleic Acid Receptors and Sensors in Acute Viral Infections

The human immune system has evolved to provide protection against a diversity of microorganisms, including viral infections. Ones of the most important factors involved in suppressing the development of a viral infection is the production of interferons via the activation of intracellular nucleic acid sensors. The endosomal TLRs (such as TLR3, 7, 8, and 9) recognize diverse endogenous danger signals as well as nucleic acid components released from uncoated virions. Activation of TLRs results in the production of type I interferons and proinflammatory cytokines, chemokines (including TNF-α, IL-6, IL-8, IP10, and others) thereby initiating inflammatory responses. In addition, the intracellular RNA sensors, such as MDA-5 (Melanoma Differentiation-Associated protein 5) and RIG-I (retinoic acid-inducible gene I) as well as the DNA sensors STING (stimulator of interferon genes) and AIM2 (absent in melanoma 2) that recognize conserved nucleic structures mediate the production of interferons and proinflammatory cytokines when activated.

### Toll-Like Receptors (TLRs)

TLRs are central to the control of innate immune responses through the recognition of pathogen-associated molecular patterns (PAMPs) and endogenous damage-associated molecular patterns (DAMPs) (9). Therefore, TLRs are not only critical for host defense against pathogens but also contribute to the pathogenesis of autoimmunity (10, 11). Several TLR agonists and antagonists are regarded as promising therapeutic agents for the treatment of sepsis, asthma, vaccine adjuvants, and autoimmunity (9). The cell surface TLRs (TLR2, TLR4, and TLR5) are activated by diverse PAMPs and DAMPs, whereas the endosomal TLRs (TLR3, TLR7, TLR8, and TLR9) recognize pathogen-derived nucleic acids and, in the context of therapeutic/experimental approaches, synthetic nucleic acids or nucleic acid mimetics. Even though TLRs can bind ligand directly, recent studies have shown that high affinity and stable interactions with ligand also require TLR-associated proteins (i.e., co-receptors such as CD14, LBP) (11).

Activation of all TLRs (except TLR3) initiates signaling via MyD88-dependent and TRIF-dependent pathways. It has been shown that activated MyD88 triggers two signaling pathways: (1) the TRAF3-TBK1-IKKε-IRF3/IRF7 pathway to induce type I interferon (IFN-α and IFN-β) expression; and (2) the TRAF6-TAK1-IKK-NFκB pathway to induce proinflammatory cytokine expression. In contrast, activated TRIF primarily triggers the TRAF3-TBK1-IKKε-IRF3/IRF7 pathway to upregulate type I interferon production.

### Cytosolic Sensors

In addition to endosomal TLRs, cells are equipped with cytosolic sensors that detect invading viruses via recognition of viral DNA and RNA. Viral RNA is recognized by members of Toll-like receptors (TLR3, TLR7/8) and RIG-I-like receptors (RIG-I, MDA5, and LGP2 (laboratory of genetics and physiology 2). In addition, viral DNA stimulate IFN production via TLR9 and intracellular DNA sensors such as STING (stimulator of IFN genes), MPYS (methionine-proline-tyrosineserine), MITA (mediator of IRF3 activation), or ERIS (ER IFN stimulator) (12, 13). Signaling pathways downstream RNA and DNA sensors induce the production of restriction factors (such as type I and type III interferons) that prevent viral replication and give rise cell-intrinsic antiviral immunity (14–16).

## C-type Lectins as Pattern Recognition Receptors in Acute Viral Infections

The superfamily of C-type lectin-like domain (CTLD)-containing carbohydrate-binding proteins comprises ~150 members that can be classified into 17 groups in humans (17). While most CTLD family receptors do not contain cytoplasmic domains capable of transducing signals, several are Syk-coupled receptors have been reported to play critical roles in host immunity against fungal and non-microbial infections (18).

### Non-syk Coupled C-Type Lectin Receptors: DC-SIGN, DC-SIGNR, MR

There is ample evidence to demonstrate the critical roles of C-type lectins in the pathogenesis of DV infection (1, 19–24). Macrophages, dendritic cells, and liver sinusoid cells are the major targets of DV in humans, where the C-type lectin receptors L-SIGN/CD299 (expressed in liver sinusoid cells), DC-SIGN/CD209 and mannose receptor/CD206 (expressed in macrophages/dendritic cells) have been shown to be crucial for DV entry into these cell types. Dengue is a mosquito-borne virus and it is interesting to note that DV generated in mosquito cells infects human cells via DC-SIGN, while DV from human dendritic cells infects human cells via L-SIGN (1, 19–24). These observations led to the hypothesis that distinct glycans associated with DV derived from human or insect cell origins may contribute the differential usage of DC-SIGN and L-SIGN in target cell infection. Despite the involvement of these C-type lectin receptors in the pathogenesis of viral infections, they do not contain intracellular motifs to enable the initiation of signaling cascades (25), nor do they transduce signals via associated adaptor proteins as observed in T cell receptors, B-cell receptors, or Syk-coupled receptors. As well as membrane-associated lectins, the soluble C-type lectin mannose binding lectin (MBL) not only binds DV generated from both human and mosquito cells, but also activates the complement system to neutralize DV infection (26).

### Syk-Coupled C-Type Lectin Receptors: CLEC5A and CLEC2

All the Syk-coupled C-type lectin receptors (CLRs) are type II transmembrane proteins which belong to group II or group V of the CTLD superfamily. Group II includes four Syk-coupled CLRs (CLEC4B/DCAR2, CLEC4C/BDCA-2, CLEC4E/Mincle, and CLEC6A/Dectin-2), whilst group V (also known as the NK receptor-like lectin family) includes CLEC2, CLEC5A/MDL-1, CLEC7A/Dectin-1, and CLEC9A/CD370 (27). Among these Syk-coupled C-type lectins, activation of CLEC5A by flaviviruses and influenza virus induces the production of inflammatory cytokines and NETosis, while these viruses also activate CLEC2 in platelets to elease extracellular vesicles (EVs), which further enhance inflammatory cytokine release and NETosis via CLEC5A and TLR2 in macrophages and neutrophils.

### CLEC5A: A Pattern Recognition Receptor in Myeloid Cells

CLEC5A, also known as myeloid DAP12-associating lectin-1 (MDL-1), is abundantly expressed in myeloid lineages, including neutrophil, monocyte, macrophage, osteoclast, microglia, and dendritic cells. While CLEC5A does not contain a cytoplasmic motif, activation of CLEC5A recruits the DAP12 adapter protein, thereby triggering downstream signaling via Syk. Structural analysis shows that human CLEC5A is an N-linked homodimeric glycoprotein expressed on cell surface (28). The ligand for CLEC5A was unknown prior to the identification of its interaction with dengue virus (1). We showed that when CLEC5A is activated by binding to the dengue virion this leads to DAP12 phosphorylation and induction of proinflammatory cytokines via Syk-mediated pathways. CLEC5A is responsible for DV-induced hemorrhagic fever (DHF) and dengue shock syndrome (DSS), which represent the most severe responses to DV infection and are characterized by plasma leakage due to increased vascular permeability. Blockade of the DV-CLEC5A interaction suppresses the secretion of proinflammatory cytokines, but without affecting interferon-α release. Furthermore, blockade of DV-CLEC5A interaction by anti-CLEC5A monoclonal antibodies (mAbs) efficiently alleviating plasma leakage and systemic hemorrhaging, thus protect mice from DV-induced shock and lethality in mice lack responsiveness to IFN–α and β (signal transducer and activator of transcription 1 (STAT1)-deficient mice). Thus, blockade of CLEC5A-mediated signaling in DV infected cells provides a promising strategy for attenuating systemic vascular leakage and increasing the survival rate of patients suffering from DHF/DSS; this approach might also be applicable to other virus-induced inflammatory diseases (1). However, even though blockade of CLEC5A protects mice from lethal doses of DV, the protective effect is ~50% at best. This suggests the presence of yet-discovered pathway in DV-induced lethality, which is the focus of ongoing research.

We have shown that DV infection induces abundant IL-1β and IL-18 production by inflammatory macrophages and also mediates pyroptosis in these cells as a consequence of upregulating pro-IL-1β, pro-IL-18 and NLRP3 and enabling caspase-1 activation; blockade of CLEC5A inhibits DV-induced NLRP3 inflammasome activation and cell death (29). We found that osteoclasts are highly susceptible to DV infection, which induces the production of cytokines and infectious virions at levels similar to macrophages and nuclear translocation of the transcription factor NFATc1, via CLEC5A. The transient inflammatory response that occurs in bone tissue during DV infection is associated with elevated osteolytic activity and release C-telopeptide of type I collagen (CTX-1) from bone. Moreover, *clec5a*^−/−^ *stat1*^−/−^ mice are resistant to DV-induced osteolysis, and anti-CLEC5A mAb inhibits DV-induced osteolytic activity and CTX-1 releases in *clec5a*^++^ *stat1*^−/−^ mice. These observations indicate that DV osteoclast and increase osteolytic activity via CLEC5A. The identification of osteoclasts as a novel target for DV provides a mechanism for some of the clinical symptoms experienced by dengue patients; i.e., as a consequence of transient upregulation of osteolysis (30).

Moreover, we demonstrated that Japanese encephalitis virus (JEV) binds CLEC5A directly to trigger the phosphorylation of DAP12 and the release of proinflammatory cytokines and chemokines in macrophages. Even though blockade of CLEC5A cannot inhibit JEV infection to neurons and astrocytes, JEV-induced cell death and proinflammatory cytokines in microglia-neurons-astrocyte mixed culture was dramatically inhibited by anti-CLEC5A mAb. This effect is achieved by blockade of JEV-CLEC5A interactions in microglia, thereby attenuating proinflammatory cytokines and toxic substances released from microglia. Furthermore, peripheral administration of anti-CLEC5A mAb not only reduces JEV-induced neuronal inflammation and lethality, but also restore blood-brain barrier (BBB) integrity after JEV infection. This observation suggests that anti-CLEC5A mAb can penetrate BBB and attenuates JEV-induced neuronal inflammation in brain, thereby restore BBB integrity and reduces neuronal toxicity and lethality. In contrast to conventional immunosuppressants, anti-CLEC5A mAb-mediated immunomodulation does not inhibit host immunity to JEV, as all surviving mice produce high titer of JEV neutralization Ab (both IgM and IgG) and develop protective cellular immunity against JEV infection. These observations indicate that CLEC5A play a critical role in JEV-induced pathological changes, and blockade of JEV-CLEC5A interactions by anti-CLEC5A mAbs is a promising strategy to brain damage and neuronal sequalae after JEV infection (31).

In addition to flaviviruses, we further find that CLEC5A interacts with the hemagglutinin protein of influenza viruses (H1N1, H5N1, H7N9), and blockade of influenza virus-CLEC5A interactions by anti-CLEC5A antibodies reduces proinflammatory cytokine production without attenuating influenza virus replication in human macrophages. Furthermore, reduced levels of TNF-α and IP-10 were found in *clec5a*^−/−^ BMDMs (bone marrow-derived macrophages) than those in wild type cells. Upon lethal challenges with a recombinant H5N1 influenza virus, lower levels of proinflammatory cytokines and less pulmonary cell infiltration with higher survival rates are noted in *clec5a*^−/−^ mice, despite comparable viral loads are noted in both WT and *clec5a*^−/−^ mice. These results indicate that influenza virus activates CLEC5A to enhance inflammatory reactions and cause severe tissue damage, and combination of current anti-viral drugs with anti-CLEC5A mAb will provide better effects to reduce tissue damage and lethality (3).

### CLEC2: A Pattern Recognition Receptor in Platelets

CLEC2 is highly expressed by platelets and megakaryocytes and low level expression of this Syk-coupled CLR has also been reported in mouse dendritic cells (32) and neutrophils (33). Human CLEC2 is a 229 amino acid type II transmembrane protein containing a putative N-linked glycosylation site in the extracellular domain. In contrast to CLEC5A, CLEC2 contains a single YxxL motif (hemITAM) in its intracellular domain, and dimerization of CLEC2 upon ligand engagement, activates platelets via Syk and SLP-76 (25, 34, 35). The O-linked glycoprotein podoplanin has been identified as the endogenous ligand of CLEC2. Podoplanin is a mucin-type transmembrane protein expressed in type I alveolar cells, kidney podocytes, and lymphatic endothelial cells (36). Activation of CLEC2 by podoplanin during embryonic development is required for blood/lymphatic vessel separation (37, 38), but although CLEC2 is involved in thrombus stabilization, its absence does not significantly increase bleeding tendency (39). CLEC2 also interacts with aggretin (rhodocytin) (40), a snake venom protein isolated from *Calloselasma rhodostoma* (41) that induces platelet activation and aggregation via its binding to CLEC2 (40). In addition to protein ligands, CLEC2 also binds to fucoidans (42), which are sulfated polysaccharides mainly comprised of fucose, but also containing other monosaccharides and uronic acid (43).

CLEC2 have been shown to capture human immunodeficiency virus (HIV) via DC-SIGN and CLEC-2, thereby facilitate viral dissemination in infected patients (44). Moreover, CLEC2 is responsible for immunothrombosis in the context of bacterial infections (45, 46). It has been reported that the absence of CLEC2 increases clinical severity in a cecal ligation and puncture (CLP) model of sepsis following injection of bacterial lipopolysaccharides (47), and deletion of CLEC2 in this model exacerbates cytokine storm and inhibits inflammatory macrophage recruitment to the infected peritoneum, resulting in increased bacterial load and organ injury (47). Deletion of CLEC2 also enhances the severity of brain inflammation in the mouse experimental autoimmune encephalomyelitis (EAE) model, where there is evidence that the podoplanin/CLEC2 axis promotes resolution of inflammatory reactions in autoimmunity (48, 49).

Recently, CLEC2 was shown to be a novel pattern recognition receptor for DV, where DV infection activates platelets to express CD62p, CD63 and to release extracellular vesicles (EVs), including microvesicles (MVs) and exosomes (EXOs) (50). We have shown that DV binds to CLEC2 on platelets, promoting the release of EVs, including EXOs (DV-EXOs) and MVs (DV-MVs). While EXOs and MVs from resting platelets do not have any activity, DV-EXOs and DV-MVs are potent “endogenous danger signals” which trigger the activation of CLEC5A and TLR2, respectively, to promote NETosis and production of proinflammatory cytokines in neutrophils and macrophages. While blockade of CLEC5A offers ~30% protection rate, simultaneous blockade of CLEC5A and TLR2 further increase mice survival rate up to 90%. These observations indicate that CLEC5A/TLR2 is not critical DV-induced pathogenesis, but also plays important roles in platelet-leukocyte interactions via recognizing platelets-derived EXOs and MVs. Thus, targeting CLEC5A/TLR2 have the potential to underpin novel strategies for treating acute viral infections.

### Heterocomplexes of C-Type Lectins

It has become clear that pathogens carry multiple PAMPs and activate immune cells via multiple receptors. For example, DV initiates inflammatory responses through activation of both CLEC5A and TLR7 associated pathways, while *Listeria monocytogenes* and *Staphylococcus aureus* activate NALP3 (NACHT, LRR and PYD domains-containing protein), NLR family NLRC4 (CARD domain-containing protein 4) and AIM2 (absent in melanoma 2) inflammasomes and proinflammatory cytokine release via CLEC5A and TLR2 (51). CLEC2 has been shown to form ligand-dependent multimers with other platelet receptors to activate inflammatory signaling pathways (52).

**Figure 1:**
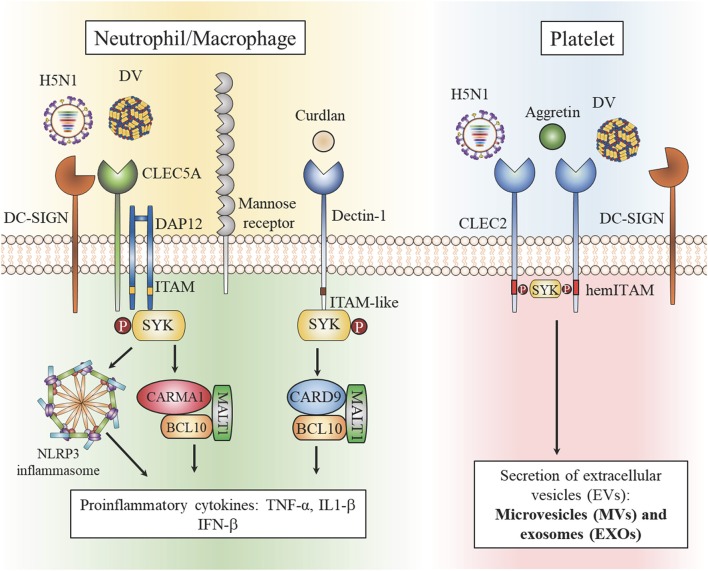
Heterocomplexes of C-type lectins in myeloid cells and platelets. Dengue virus (DV) and influenza virus (H5N1) are captured by the high affinity receptors DC-SIGN and mannose receptor (MR). The formation of heterocomplexes enables Syk-mediated signaling via low affinity CLEC5A to activate the NALP3 inflammasome and induce the formation of CARMA1/BCL10/MALT1, thereby upregulating proinflammatory cytokine production (right). DV and H5N1 are captured by the high affinity receptor DC-SIGN and receptor multimerization leads to activation of the low affinity receptor CLEC2 to stimulate production of extracellular vesicles (EVs). Aggretin, a high affinity ligand for CLEC2, induces EV release and platelet aggregation independently of DC-SIGN (left).

Viral glycans contain multiple terminal sugars, including mannose, fucose, sialic acids with or without sulfation; therefore, it is not surprising that multiple lectin receptors on host cells colocalize during engagement with these PAMPs. It has been demonstrated that DV interacts with CLEC5A, DC-SIGN (dendritic dell-specific intercellular adhesion molecule-3-grabbing non-integrin), DC-SIGNR (1), and mannose receptor (MR) (24). Although DV binds with much lower affinity to CLEC5A than to DC-SIGN or DC-SIGNR, only CLEC5A has been clearly shown to mediate downstream signaling pathways after engagement with DV. DV-induced activation of CLEC5A is dependent on DC-SIGN and MR (53) and imaging analysis has revealed that engagement of DV with myeloid cells triggers colocalization of CLEC5A and MR/DC-SIGN to form a hetero-multivalent complex (53). The lectin heterocomplex would facilitate the formation of multivalence interactions between viral glycans and C-type lectins with distinct glycan-binding affinity to enable signaling via CLEC5A. Even though the interaction between DV and CLEC2 is weak (54), DV also binds platelets via DC-SIGN (55). Thus, DV may also trigger the formation of DV-CLEC2-DC-SIGN complex to enable signaling via CLEC2 (Figure 1).

## Heterocomplexes of CLEC5A and TLR2 in Bacterial and Viral Infections

In addition to the formation of heterocomplexes between C-type lectins, we have shown that engagement of *L monocytogenes* with macrophages and neutrophils induces the formation of CLEC5A/TLR2 heterocomplexes. While TLR2 binds lipoteichoic acid from the *L. monocytogenes* cell wall, CLEC5A binds to GlcNAc-MurNAc disaccharides within the backbone of bacterial wall teichoic acids (51). Coactivation of CLEC5A and TLR2 by *L. monocytogenes* stimulates the production of inflammasomes and induces NET formation, proinflammatory cytokine expression and γδ CD4^+^ Th17 cells differentiation (51). In contrast to viral infection, CLEC5A-deficient mice are highly susceptible to infections with bacteria including *L. monocytogenes* and *S. aureus* (51).

**Figure 2:**
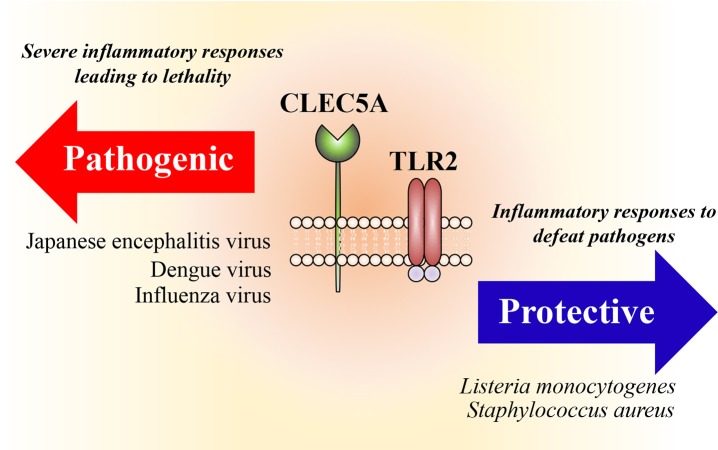
Heterocomplexes of CLEC5A and TLR2. Activation of CLEC5A/TLR2 heterocomplexes promotes formation of inflammasomes (NALP3, NLRC4, AIM-2) and induces NET formation and proinflammatory cytokine release. While this is beneficial to the host to fighting bacterial infections, overactivation of CLEC5A/TLR2 is detrimental during acute viral infections.

Whilst the host immune system is beneficial in promoting the clearance of many pathogens it can also have detrimental effects in some contexts (Figure 2). One of the most cited examples is the high susceptibility to bacterial infection after acute viral infections, for example with influenza virus (56). Potential explanations for this are the association of NET formation with lung injury (57), and the central role of inflammasome activation in viral pathology (58); blockade of CLEC5A/TLR2 attenuates NET formation and inflammasome activation and is thus beneficial to the host during acute viral infection (50). Moreover, excessive NETs cause damages of epithelium and vascular endothelium during bacterial infections, promotes vascular occlusion and tumor cell metastasis, and enhances autoantibody production (59). Because CLEC5A is also responsible for ConA-induced shock syndrome (60) and synovial inflammation in collagen-induced arthritis (61), thus bloackade of CLEC5A/TLR2 may reduce bacteria-induced systemic permeability change, inhibit tumor metastasis, and attenuate tissue damages during aseptic inflammation.

## Conclusions

Following our identification of CLEC5A as the pathogenic host factor in flaviviruses, we further found that CLEC2 and TLR2 are pathogenic receptors in DV, JEV, and H5N1 infections. These viruses are captured by high affinity receptors (DC-SIGN and MR) to induce inflammatory cytokine release via activation of CLEC5A and CLEC2. We have also demonstrated the complex interactions between platelets and leukocytes during viral infection. These studies have demonstrated the molecular mechanisms underlying virus-induced cytokine storms and lethality and revealed a new direction for the development of treatments for acute viral infections. Moreover, excessive NETs contribute to the pathogenesis of autoimmune diseases (62), where the risk of developing autoimmune diseases is increased in patients post DV infection (63). Thus, blockade of CLEC5A and TLR2 using a bi-specific anti-CLEC5A/TLR2 mAb has the potential to reduce the risk of autoimmunity post-DV infection, and have therapeutic effects in autoimmune diseases by limiting excessive NET formation and persistent inflammasome activation.

## Perspective

There has been a tendency to investigate host responses to pathogen from the perspective of single PAMP-receptor interactions with a focus on endosomal TLRs and cytosolic sensors that recognize highly conserved DNA and RNA structures. However, the complex structures of viruses contain a diversity of PAMPs that stimulate multiple receptors on host immune cells and modulate innate immune responses to individual viruses. Furthermore, viruses frequently infect multiple cell linages, including both immune and non-immune cells, and induce cell-cell interactions during acute viral infections. Our previous studies demonstrated virus-mediated activation of platelets to enhance NET formation, proinflammatory cytokine release, and inflammasome activation via membrane receptors that include CLEC5A. This supported the idea that host cells not only recognize conserved ribonucleic acid structures to stimulate production of interferons, but also recognize viral membrane components and extracellular vesicles to further promote multiple innate immune pathways.

The ability of DV-EVs to enhance inflammatory reactions and NETosis suggest that activated EVs can be regarded as novel “endogenous danger signals” or “damage-associated molecular patterns” which activate innate immunity via CLEC5A and TLR2. In this regards, CLEC5A/TLR2 heterocomplex may play important roles in the pathogenesis of aseptic inflammations. This argument is further supported by the observation that CLEC5A^+^ cells are responsible for collagen-induced autoimmune arthritis (61) and ConA-induced lethal shock syndrome (60). It would be interesting to test whether EV-CLEC5A/TLR2 interactions contribute to the pathogenesis of aseptic inflammation and autoimmune diseases in the future.

In addition to mediating acute infections, some viruses interact with innate immune receptors to establish chronic infections. In this context innate immune receptors act as “pathogenic host factors” that transduce inhibitory signals after binding virus. Human hepatitis B virus (HBV) frequently causes chronic and persistent infections following initial inflammatory responses. HBV is an enveloped DNA virus that infects hepatocytes and causes persistent infection due to the presence of covalently closed circular (ccc)DNA, which becomes inserted into the host genome in hepatocytes. The HBV envelope contains large, middle, and small HBV surface antigens (HBsAgs); the large HBsAg is present on virions, known as Dane particles, that contain the viral genome and are able to infect and replicate after entering hepatocytes, but this is not the case for the middle and small HBsAgs. Interestingly, the middle and small HBsAgs outnumber the large HBsAg and it has been speculated that these un-infectious HBsAgs may suppress host immunity and inhibit the production of anti-HBsAg Ab. Identification of “inhibitory” innate immunity receptor(s) for HBV may provide an answer the un-resolved questions surrounding chronic persistent HBV infection and further our understanding of the roles of viral membrane components in the complex pathogenesis of viral infections.

## Author Contributions

P-SS: discussion and figure drawing. S-LH: organization and writing of manuscript.

### Conflict of Interest

The authors declare that the research was conducted in the absence of any commercial or financial relationships that could be construed as a potential conflict of interest.
